# Tracking progressive pathological and functional decline in the rTg4510 mouse model of tauopathy

**DOI:** 10.1186/s13195-017-0306-2

**Published:** 2017-09-20

**Authors:** Thomas Blackmore, Soraya Meftah, Tracey Karen Murray, Peter James Craig, Anthony Blockeel, Keith Phillips, Brian Eastwood, Michael J. O’Neill, Hugh Marston, Zeshan Ahmed, Gary Gilmour, Francois Gastambide

**Affiliations:** Lilly Research Laboratories, Eli Lilly & Co. Ltd., Windlesham, UK

**Keywords:** Alzheimer’s Disease, Tau, Neurodegeneration, rTg4510, Behaviour, Cognition, Pathology

## Abstract

**Background:**

The choice and appropriate use of animal models in drug discovery for Alzheimer’s disease (AD) is pivotal to successful clinical translation of novel therapeutics, yet true alignment of research is challenging. Current models do not fully recapitulate the human disease, and even exhibit various degrees of regional pathological burden and diverse functional alterations. Given this, relevant pathological and functional endpoints must be determined on a model-by-model basis. The present work explores the rTg4510 mouse model of tauopathy as a case study to define best practices for the selection and validation of cognitive and functional endpoints for the purposes of pre-clinical AD drug discovery.

**Methods:**

Male rTg4510 mice were first tested at an advanced age, 12 months, in multiple behavioural assays (step 1). Severe tau pathology and neurodegeneration was associated with profound locomotor hyperactivity and spatial memory deficits. Four of these assays were then selected for longitudinal assessment, from 4 to 12 months, to investigate whether behavioural performance changes as a function of accumulation of tau pathology (step 2). Experimental suppression of tau pathology—via doxycycline administration—was also investigated for its effect on functional performance.

**Results:**

Progressive behavioural changes were detected where locomotor activity and rewarded alternation were found to most closely correlate with tau burden and neurodegeneration. Doxycycline initiated at 4 months led to a 50% suppression of transgene expression, which was sufficient to prevent subsequent increases in tau pathology and arrest related functional decline.

**Conclusions:**

This two-step approach demonstrates the importance of selecting assays most sensitive to the phenotype of the model. A robust relationship was observed between pathological progression, development of phenotype, and their experimental manipulation—three crucial factors for assessing the translational relevance of future pre-clinical findings.

## Background

The vast societal and economic burden of Alzheimer’s disease (AD) represents a growing problem [1]. Despite extensive effort, AD drug discovery programmes have so far lacked late-stage clinical success [2, 3]. While some of these failures have arisen from incomplete understanding of drug properties or inappropriate study design [4, 5], many may also reflect true negative effects. Given the several hundred interventions reported to mitigate pathological and behavioural alterations in AD mouse models, it is reasonable to question the predictive validity of early AD drug discovery [6]. Pre-clinical validation forms an integral part of nearly every drug project, yet justification of disease model as well as the endpoint(s) chosen often appears ad hoc, or simply lacking. In contrast, clinical efficacy of AD therapies is currently based on pre-defined cognitive test batteries and activities of daily living questionnaires. Therefore, a major consideration for future AD pre-clinical efforts would be to better understand the benefits and limitations of existing behavioural and functional assays, their relationship with disease progression (e.g. [7]), and ultimately with clinical endpoints.

Contemporary research suggests tau rather than amyloid pathology is most closely associated with neurodegenerative processes [8, 9] as well as cognitive and functional decline, prompting a shift towards tau-based drug discovery [10]. Whilst current mouse models are unable to precisely recapitulate AD-like anatomical, temporal, and spatial progression of tau pathology, they still provide powerful tools for exploring disease-modifying therapies and are widely used in AD drug discovery [11–13]. One of the most widely used models of tauopathy is the repressible rTg(tet-o-TauP301L)4510 (or rTg4510) mouse [14, 15]. A significant advantage of this model is that expression of mutant tau is controllable by doxycycline administration, offering an ideal experimental positive control to determine functional effects of suppression of tau pathology. While the progression of tau pathology has been characterised thoroughly in this model [14, 15], functional decline has often been described using cross-sectional comparisons of one or two endpoints; these vary from study to study and with very little justification of why these assays were selected.

The present work aims to extend the initial phenotypic characterisation of the rTg4510 mouse model [15] and offer a detailed approach to the selection and validation of behavioural endpoints relevant for AD drug discovery. Male rTg4510 mice were first tested in seven different behavioural assays at an advanced pathological stage, between 12 and 15 months of age; the hypothesis was that rTg4510 mice would display robust and selective alterations in locomotor activity and spatial memory. The behavioural assays most sensitive to tau pathology were then selected and used in a separate and larger cohort of mice to assess longitudinally, from 4 to 12 months, the capacity of these endpoints to track progressive functional decline. The sensitivity of these functional endpoints to experimental modulation of tau pathology was tested in parallel via the administration of doxycycline from 4 months of age in a subgroup of rTg4510 mice. The hypothesis was that doxycycline would arrest the progression of tau burden and atrophy and thereby contribute to maintaining behavioural capacity at baseline levels, strengthening the hypothesis that such functional endpoints could be used to support future tau-based drug studies.

## Methods

### Subjects and study design

The rTg(tet-o-TauP301L)4510 bi-transgenic mouse model [14, 15] was licensed from the Mayo Clinic (Jacksonville, FL, USA), bred on a mixed FVB/NCrl + 129S6/SvEvTa background by Taconic (Germantown, MD, USA), and delivered to Eli Lilly and Company (Windlesham, UK) via Envigo (Loughborough, UK). All mice were subjected to health screening, tested positive for non-MRSA *Staphylococcus aureus* and *Proteus* bacteria, and treated accordingly for 10 days with Baytril (NVSL, Stoke-on-Trent, UK) before experimental use. All subsequent animal procedures were carried out at Eli Lilly and Company Limited, in accordance with the UK Animals (Scientific Procedures) Act 1986 and with approval of the local Animal Welfare and Ethical Review Board. Mice were housed on a 12 h light/dark cycle with water provided ad libitum, and maintained on a food-restricted diet at no less than 85% of their free-feeding body weight. Due to known sex differences in both the onset and severity of tau pathology in rTg4510 mice [16], only male mice were used for the present study. Note that all female mice were used for other studies.

Two cohorts of male rTg4510 mice were used for these studies (see Table 1). The first cohort consisted of nine bi-transgenic rTg4510 (CC) mice and nine wild-type littermate controls (WW). These mice were tested between 12 and 15 months of age in a wide range of behavioural assays. The second cohort consisted of 93 CC mice and 30 WW controls. Mice were tested longitudinally, every other month from 4 to 12 months of age, in the open-field locomotor activity and T-maze rewarded alternation assays. At 4-, 8-, and 12-month time points, a subset of mice—representative of average behavioural performance of their respective treatment groups—were tested in two additional behavioural assays (Y-maze spatial novelty preference and aversive swim escape Y-maze spatial reference memory tasks) and finally perfused for pathological assessments. Y-maze assays could only be tested at these time points, and so were not tested longitudinally due to the chance that repeated testing may evoke confounding practice effects, coupled with a level of labour intensity of testing that significantly restricts throughput. Brain samples from animals at 8- and 12-month time points also underwent quantitative reverse transcription polymerase chain reaction (RT-qPCR). The doxycycline treatment was initiated at 4.1 months, following initial open-field locomotor activity and T-maze rewarded alternation testing. To assign animals to doxycycline treatment groups, T-maze performance of animals from the 4-month time point was ranked and then a random number generator was used to generate a sequence which allocated animals to either the doxycycline (CC + dox) or vehicle-treated (CC) control group. Re-assignment of six animals was required to ensure all animals within a cage received the same treatment. CC + dox mice received a bolus of 10 mg/kg q.d. doxycycline hyclate (Sigma Aldrich, Dorset, UK) via oral gavage for the first 2 days of treatment and were then maintained on doxycycline-mixed chow diet (Harlan Teklad Rodent Diet; 200 mg doxycycline per kg of dietary chow) until the end of the study. CC and WW control groups received a bolus of a 5% glucose vehicle (10 ml/kg) via oral gavage for the first 2 days of treatment and were then maintained on standard chow. The dietary composition of doxycycline-mixed and standard chow was similar for each group.

**Table 1:** Summary of behavioural and pathological endpoints investigated in the two cohorts of male rTg4510 mice

Cohort 1					12-15 months
Behaviour: spontaneous Y-maze continuous alternation, locomotor activity, Rotarod, aversive Y-maze spatial reference memory, rewarded T-maze alternation, Y-maze spatial novelty preference and rewarded Y-maze visual discrimination.					CC_(9)_ WW_(9)_
					**↓**
Pathology: immunohistochemistry					Perfused
Cohort 2	4 months	6 months	8 months	10 months	12 months
Locomotor activity	CC_(93)_ WW_(30)_	CC-d_(37)_ CC_(38)_ WW_(19)_	CC-d_(36)_ CC_(37)_ WW_(19)_	CC-d_(19)_ CC_(18)_ WW_(9)_	CC-d_(19)_ CC_(18)_ WW_(9)_
Rewarded T-maze alternation	CC_(93)_ WW_(30)_	CC-d_(37)_ CC_(38)_ WW_(19)_	CC-d_(36)_ CC_(37)_ WW_(19)_	CC-d_(19)_ CC_(18)_ WW_(9)_	CC-d_(19)_ CC_(18)_ WW_(9)_
	↓		↓		↓
Y-maze spatial novelty preference	CC_(16)_ WW_(11)_		CC-d_(17)_ CC_(18)_ WW_(10)_		CC-d_(19)_ CC_(18)_ WW_(10)_
Aversive Y-maze spatial reference memory	CC_(16)_ WW_(11)_		CC-d_(17)_ CC_(18)_ WW_(10)_		CC-d_(19)_ CC_(18)_ WW_(10)_
	↓		↓		↓
Immunohistochemistry and RT-qPCR	Perfused		Perfused		Perfused

12-month-old male rTg4510 mice from the first cohort were tested in seven behavioural assays, as ordered in the table, and then perfused for immunohistochemical assessment of tau pathology

Male mice from cohort 2 were tested from 4 to 12 months in the four pre-defined assays. Following behavioural testing in locomotor activity and T-maze alternation at 4 months, CC animals were split into two treatment groups: doxycycline (CC-d) and non-doxycycline (CC) and tested longitudinally every other month in these two assays until 12 months. At 4, 8, and 12 months, subsets of animals were tested in the Y-maze spatial reference memory and novelty preference assays after which they were all perfused for immunohistochemistry and RT-qPCR analysis

Arrows indicate animals removed from the longitudinal study and undergoing further behavioural testing and perfusion

_(n)_ = number of mice per group at time point

*CC* non-doxycycline treated bi-transgenic rTg4510, *CC-d* doxycycline-treated bi-transgenic rTg4510, *RT-qPCR* reverse transcription quantitative polymerase chain reaction, *WW* wild-type rTg4510

### Behaviour

#### Motor activity and motor co-ordination tasks

##### Open-field locomotor activity

Spontaneous locomotor activity was assessed in clear Perspex open-field arenas (40 × 40 × 30 cm) under complete darkness. Arenas were based on infrared fields (100 × 100 cm) with four boxes placed on each field and monitored using overhead infrared cameras (Sanyo VCV-3412P, Tracksys Ltd., UK). Cameras fed into a Quad compressor unit (Sanyo VDM-801P, Tracksys Ltd., UK) which relayed data to a computer running the image analysis software Ethovision XT v8.5 (Noldus, Netherlands). Mice were allowed to freely explore the arena for 60 min during which time the distance moved was measured as a total over 60 min (primary outcome measure) and in 5-min time bins to discern progressive changes in activity/habituation (secondary outcome measure).

##### Rotarod

Motor co-ordination was assessed in a five-station mouse Rotarod (ENV 575MA, Med Associates Inc., USA). Mice were placed on a slowly rotating drum and their ability to remain on the drum was assessed for up to 5 min. An accelerating speed protocol was utilised whereby rotation speed gradually increased from 4 rpm to a maximum of 40 rpm over the 5-min test period. Fall latency (primary outcome measure) was recorded over three separate trials and is reported here as an average of the three trials. There was an inter-trial interval (ITI) of approximately 5–10 min to allow mice to recover. In some cases, mice were able to cling to the rotating beam for one full revolution; this was registered as a fall by the experimenter and the trial was ended.

#### Short-term habituation and spatial working memory tasks

##### Y-maze spontaneous, continuous alternation

Y-maze arenas were based on infrared fields (100 × 100 cm) and movement data captured as described in the open-field locomotor activity test. Each Y-maze consisted of three equal arms (30 × 8 × 20 cm), spaced 120° apart. Arms were assigned arbitrary labels of A, B, and C. Test sessions were 15 min, and began as soon as the mouse was placed in the arena. Mice were placed in the distal end of one arm, facing away from the centre of the maze and allowed to freely explore all arms of the Y-maze. Initial data acquisition was conducted by Ethovision XT v8.5 (Noldus, Netherlands) and then subject to further processing by in-house software (Ethovision Collection, Handling and Ordering v2.2.0.0, Lilly Research Laboratories, UK). The number of arm entries and non-repeating arm entry triads (e.g. ABC, CAB) were recorded, from which percentage alternation (primary outcome measure), as well as total distance moved (secondary outcome measure) were derived.

##### Y-maze spontaneous, spatial novelty preference

Y-maze arenas, infrared fields, and the video tracking system used were identical to those described for the continuous alternation task. Spatial novelty preference was assessed in two separate phases, an exposure phase and a test phase. Mice were pseudo-randomly assigned two arms (the “start arm” and the “other/familiar arm”) to which they were exposed and allowed to freely explore for 5 min (“exposure” phase). The entrance to the third “novel” arm was blocked by the presence of a large opaque insert. At the end of the 5-min period, the mouse was removed from the maze and placed back in its home cage for 1 min, following which the “test” phase started. The insert was removed; mice were placed back into the start arm and allowed to explore the entire maze (i.e. all three arms) for 2 min. Videos were recorded and manually scored offline using Abacus v2.0 (Lilly Research Laboratories, UK). A discrimination ratio of the time spent in the novel arm (primary outcome measure: time in novel arm/(time in novel + familiar arms)) was calculated for the test phase. The amount of time spent in each arm as well as the number of arm entries (co-secondary outcome measures) were recorded during both exposure and test phases.

##### T-maze rewarded alternation

Discrete-trial rewarded alternation was tested using a semi-automated T-maze (Apogee Engineering Analysis Solutions, Norwich, UK). This apparatus was constructed of matte black, 8-cm wide Perspex with 20-cm high transparent Perspex walls. The external lengths of maze edges were 86 cm (choice end), 105 cm (return arm) and 22.5 cm (delay end). The centre arm was 83 cm in length and a door was located 63 cm from the choice point forming a holding area at the base of the start arm. The entry of an animal into specific areas of the maze was detected using infrared beam breaks and passed to a microcontroller (Arduino Mega 2560). Custom-made Matlab programs automatically controlled the maze doors and test procedure, allowing it to run without human intervention. Rewards were delivered by three pellet dispensers, one located at the end of each reward arm and a third in the delay/holding area of the maze to encourage return of the animal to the starting point for the subsequent test phase or trial. Mice were trained following a two-stage protocol: forced alternation training and discrete-trial rewarded alternation testing. During the forced alternation training stage, mice were released from the holding area at the base of the T-maze, allowed to run along the centre-arm and forced to turn toward one of the reward areas to receive a sucrose pellet reward. Mice then returned to the holding area to collect a second reward pellet, after which another forced trial was initiated. At this stage, a 0-s ITI was used, and mice were trained for a maximum of 60 trials or 30 min daily. Once most mice were performing 40 training trials or more in a session, mice were moved on to the discrete-trial rewarded alternation protocol. Each trial consisted of two phases—a sample phase and a test phase. During the sample phase, mice were forced to turn toward the left or right arm and return to the starting/holding area. Two reward pellets were collected along the way, one at the end of the choice arm and one in the holding area. During the test phase of each trial, mice were allowed to choose between the two arms of the T-maze but were rewarded when visiting the novel arm only (i.e. arm not explored during the sample phase). Forced left or right allocations during the sample phase were pseudo-randomized with no more than three consecutive sample runs to the same side. Mice were allowed to run for a maximum of 20 trials or 30 min daily over 3 days of testing. A 2-s ITI and a 5-s sample-to-test delay were used. The percentage of correct choices (primary outcome measure: number of correct choice/number of trials) as well as the choice latency (secondary outcome measure) were recorded and calculated for each animal.

#### Spatial reference memory task and visual discrimination control task

##### Aversive (swim escape) Y-maze spatial reference memory

Y-maze arenas were identical to those used for continuous alternation and spatial novelty preference tasks. Extra-maze cues were placed around the room. An escape platform (8 × 8 × 11 cm) was placed at the end of one arm (i.e. the goal arm) and the maze was flooded to a depth of approximately 12 cm so that the platform was submerged. White tempera powder paint (Educational Colours Pty Ltd., Australia) was added as an opacifier to ensure the platform was not visible. Spatial reference learning was assessed over 8 days of testing, with five trials per day and an ITI of approximately 5–10 min. Mice were pseudo-randomly assigned a goal arm, defined according to its allocentric position relative to the room cues and therefore constant during the entire testing phase. Goal arms were counterbalanced with respect to treatment group such that approximately equal numbers of mice were trained to each of the three arms. The other two arms or start arms were used in a pseudo-randomised order; these were counterbalanced to ensure that, over a 10-trial block, each start arm was used five times and no more than three times consecutively. For each trial, mice were gently placed at the distal end of either start arm, facing away from the centre of the maze and allowed to swim freely to either of the other two arms. A correct choice was scored when mice entered the goal arm, after which they were allowed to spend 30 s on the escape platform before being removed from the maze. If mice chose incorrectly and entered the other arm, they were punished with 30 s of forced swim, during which entry to the goal arm was blocked. Arm entry was classed as all four paws crossing into an arm. Mice who failed to make a choice after 60 s had elapsed were removed from the maze and the trial was skipped. Twenty-four hours after the final day of spatial learning, a probe test was conducted to test for spatial reference memory. The escape platform was removed from the arena. Mice were placed in one of the two start arms and allowed 30 s of free exploration of the maze, starting when mice exited the start arm. The percentage of correct choices and percentage time spent in the goal arm (co-primary outcome measures) were calculated for the spatial learning and memory probe test, respectively.

##### Y-maze rewarded visual discrimination

Y-maze arenas used were identical to that used for the previous Y-maze tasks. For each trial, the Y-maze had a start arm and two goal arms made visually distinct by adding floor inserts; one painted grey and one black and white striped. An opaque white food well was placed at the end of each of the three arms. Mice were first familiarized to the maze without floor inserts and in a separate room to the testing room. Multiple sucrose pellets were placed in each of the food wells. Mice were left to explore the maze until they readily ate all pellets. Following this, mice were trained to discriminate between the two floor inserts to locate the goal/rewarded arm over 10 days of testing and 10 trials per day, with an ITI of approximately 10 min. Mice were pseudo-randomly assigned either one of the two patterns as a goal arm in a counterbalanced manner between groups. The start arm as well as the left/right location of the floor inserts and thus the goal arm location varied from trial to trial (i.e. there was no spatial solution), and was determined according to a pseudo-random sequence (with equal numbers of right/left presentation and no more than three consecutive trials with the same correct location). The maze was rotated 120° clockwise at random intervals, making sure spatial and olfactory cues could not reliably be used to solve the task. For each trial, the food well in the goal arm was baited with a sucrose pellet reward, and mice were placed at the distal end of the start arm, facing outwards. A correct choice was scored when mice entered the assigned/correct goal arm directly. If mice chose incorrectly they were immediately removed from the maze and then returned to their home cage. A choice was classed as all four paws being placed in either of the non-start arms. The percentage of correct choices (primary outcome measure) was calculated and used as an index of discrimination learning.

### Pathology

#### Perfusion and brain harvesting

Animals were terminally anaesthetised with pentobarbital administered via intraperitoneal injection and transcardially perfused with saline. Brains were then removed and weighed before 10–20 mg of rostral cortex tissue was dissected and snap-frozen in RNase-free tubes for RT-qPCR analysis. The remaining brain tissue was immersion fixed in 10% buffered formalin for immunohistochemical analyses

#### Quantitative reverse transcription PCR

Expression of the transgenic tau was analysed in samples taken at 8 and 12 months by RT-qPCR following standard techniques. Total RNA was isolated from snap-frozen samples of cortex using the Ambion RNAqueous 4 PCR kit (Thermofisher Scientific, UK) and following the manufacturer’s protocol; DNAse digestion was performed to remove any contaminating DNA. RNA was quantified using a Nanodrop spectrophotometer (Thermofisher Scientific, UK) and its quality assessed by running an aliquot on an Agilent Nano 6000 Chip on an Agilent 2100 Bioanalyzer (Agilent Technologies, Stockport, UK). cDNA was synthesised from 36 ng of RNA using random hexamer primers (2.5 μM) and Moloney Murine Leukemia Virus Reverse Transcriptase (1.25 U/μl) in reaction buffer containing dATP, dCTP, dTTP, dGTP (0.5 μM of each), MgCl_2_ (5.5 mM), and RNase inhibitor (0.4 U/μl) (all reagents from Thermofisher Scientific, UK). The reverse transcription was incubated at 25 °C (10 min), 48 °C (30 min), and 95 °C (5 min). Primers for qPCR were synthesised by Eurofins Genomics (Ebersberg, Germany) according to the sequences described by Santacruz et al. [14]: human tau transgene: forward 5’-CCC AAT CAC TGC CTA TAC CC-3’; reverse 5’-CCA CGA GAA TGC GAA GGA-3’; GAPDH: forward 5’-TGG TGA AGC AGG CAT CTG AG-3’; reverse 5’-TGC TGT TGA AGT CGC AGG AG-3’; mouse tau: forward 5’-AGC CCT AAG ACT CCT CCA-3’; reverse 5’-TGC TGT AGC CGC TTC GTT CT-3’. Quadruplicate qPCR reactions were assembled and aliquoted robotically into a 96-well reaction plate such that each 5-μl reaction contained 2.5 μl SYBR® Green mastermix (QuantiTect SYBR® Green PCR Kit, Qiagen, Manchester, UK), 225 pg cDNA, and appropriate primer as follows: 400 nM transgenic tau; 200 nM mouse tau; 200 nM GAPDH. The qPCR reaction was run on an ABI 7900HT Fast Real-Time PCR System using a two-stage anneal and elongation protocol with the following parameters: 50 °C 2 min, 95 °C 10 min; 95 °C 15 s, 58 °C 1 min (40 cycles); 95 °C 15 s, 58 °C 15 s, 95 °C 15 s. Data were analysed using the ∆∆Ct method using GAPDH as the reference gene and the non-doxycycline treated group as the calibrator. These analyses were performed in a blinded fashion.

#### Immunohistochemistry

Brain samples were processed using the Tissue TEK VIP processor (GMI Inc., Ramsey, MN, USA) before being embedded in paraffin wax for coronal brain sectioning. Serial sections (6 μm) were taken using HM 200 and HM 355 rotary microtomes (Thermo Scientific Microm, Germany). Immunohistochemistry (IHC) was performed using a primary antibody for tau phosphorylated at serine 409 (PG-5, 0.11 μg/ml from Peter Davies; Albert Einstein College of Medicine, Bronx, NY, USA) as previously described [17]. Stained sections were digitised using the Scanscope XT slide scanner (Aperio, CA, USA) at 20× magnification. Imagescope software (version 11.1.2.760; Aperio) was used to view the digitized tissue sections and delineate the regions of interest (ROIs) which included the hippocampus and cortex. PG-5 positive tau pathology was quantified in these ROIs using a positive pixel algorithm that was calibrated to detect only intensely labelled tau pathology, to avoid any non-specific staining. The burden of tau pathology was expressed as percentage area, and the ROI area (mm^2^) was used as a marker of atrophy. These analyses were performed in a blinded fashion.

### Statistics

All datasets were checked for normality and homogeneity of variance and transformed as appropriate, before subsequent statistical analyses were conducted using Statistica v13.0 (Statsoft Ltd., Bedford, UK). Analysis of variance (ANOVA) was used to analyse behavioural and pathological data. One-way ANOVA was used for all the cross-sectional assessments. Repeated measures ANOVA was used for analysis of the longitudinal assessments, using Proc Mixed of the SAS system (SAS/STAT v14.1, SAS Institute Inc., USA). Genotype/treatment group (WW, CC, and CC + dox) was used as the main between-subject factor, and age, time and trial block were used as within-subject factors as appropriate. Significant main effects or interactions were subsequently explored with appropriate post hoc analyses and *p* values corrected for multiple comparisons. Spearman’s rank correlation coefficient analyses were used to assess the relationship between effects on pathology vs. behaviour, as well as pathology vs. pathology and behaviour vs. behaviour. Correlation coefficients and unadjusted *p* values are reported.

## Results

### Study 1: Selection of cognitive and behavioural endpoints in 12- to 15-month-old male rTg4510 mice

#### Missing and excluded data

Four mice (three CC and one WW) were excluded from the analysis of T-maze rewarded alternation data as these animals failed to complete a minimum of five trials. One mouse (CC) was removed from spatial novelty preference analysis as it failed to leave the start arm and explore the rest of the maze during the 5-min exposure phase.

#### Severe tau pathology and brain atrophy in old male rTg4510 CC mice

**Fig. 1:**
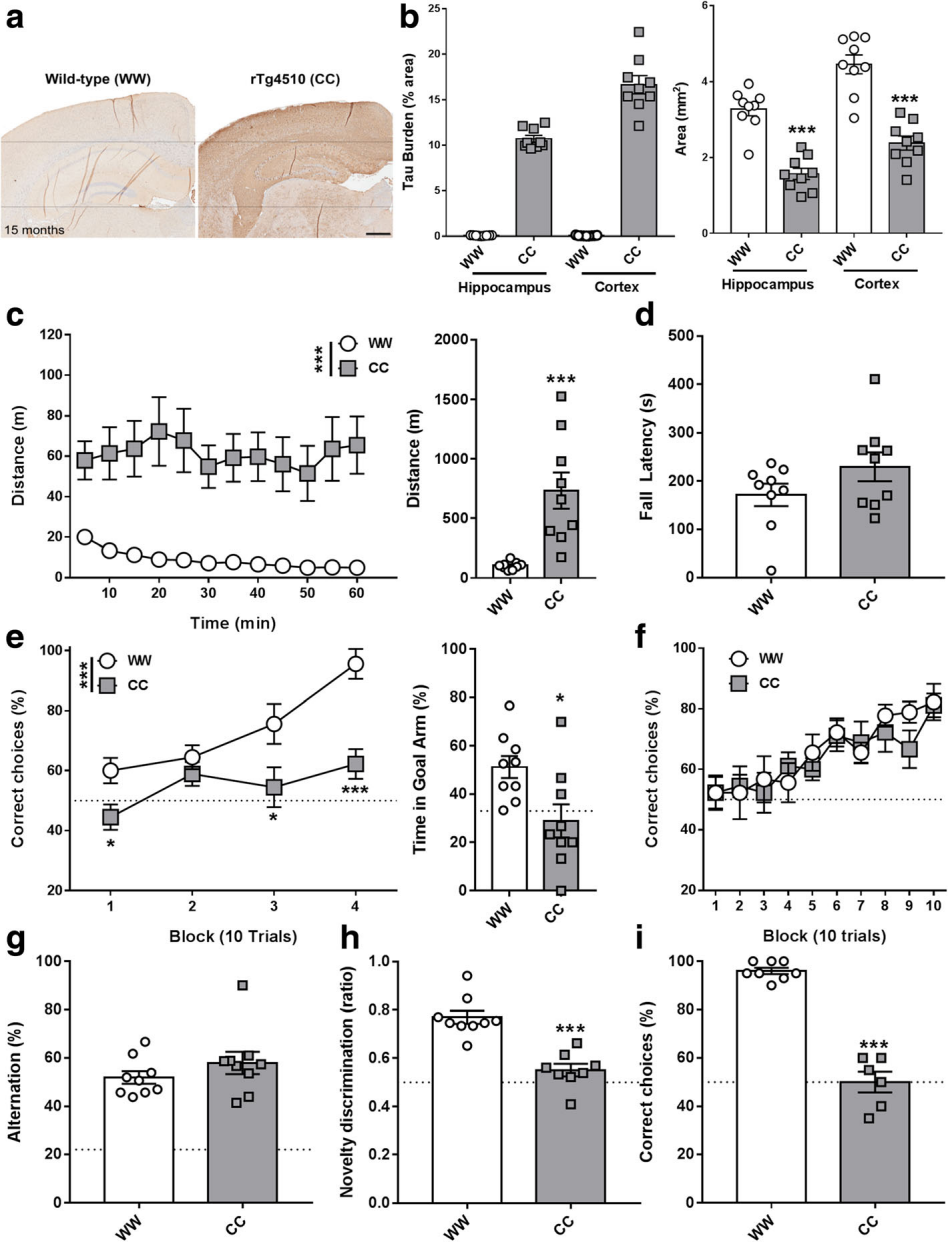
Immunohistochemical and behavioural profiling of 12- to 15-month-old rTg4510 mice. Pathology: Bi-transgenic rTg4510 (*CC*) mice displayed severe hippocampal and cortical tau burden and atrophy (**a**), as measured by PG-5-positive staining and area, respectively (**b**). Behaviour: CC mice displayed profound hyperactive behaviour in the open-field locomotor activity task (**c**), whereas motor co-ordination remains intact as measured in the Rotarod task (**d**). CC mice were impaired in the acquisition and 24-h probe testing in the swim escape Y-maze spatial reference memory task (**e**). Acquisition of a Y-maze non-spatial, visual cue discrimination learning task was no different than in wild-type/non-transgenic rTg4510 (*WW*) controls (**f**). No deficit was observed in the Y-maze continuous alternation task (**g**), while CC mice were impaired in both the spatial novelty preference (**h**) and discrete-trial rewarded alternation (**i**). *Scale bar* = 500 μm. All data are presented as mean ± SEM, *dotted lines* denote chance level. *p < 0.05, **p < 0.01, ***p < 0.001, versus WW controls

Widespread tau pathology was observed in coronal brain sections taken from 15-month-old male rTg4510 CC mice (Fig. 1a). PG-5-positive tau pathology was severe in the hippocampus (10.7 ± 0.3% burden) and cortex (16.6 ± 0.7%) of CC mice (Fig. 1b). Extensive atrophy was also observed, as indicated by significant reductions in the hippocampal and cortical area compared to age-matched WW littermate controls (hippocampus: F_1,16_ = 55.26, *p* < 0.001; cortex: F_1,16_ = 44.57, *p* < 0.001).

#### Altered motor activity but intact motor co-ordination in old male rTg4510 CC mice

Old CC mice were consistently more active than age-matched WW controls over 60 min of open-field spontaneous exploration, exhibiting no habituation over this time period (Fig. 1c; genotype: F_1,16_ = 66.71, *p* < 0.001; time: F_23,368_ = 11.15, *p* < 0.001; genotype × time: F_23,368_ = 7.45, *p* < 0.001). Despite this profound hyperactive behaviour, motor co-ordination of CC animals remained intact as demonstrated by the comparable performance between genotypes on the Rotarod task (Fig. 1d; genotype: F_1,15_ = 1.25, *p* = 0.28).

#### Impaired spatial reference memory but not visual cue discrimination in old male rTg4510 CC mice

Spatial reference learning was severely impaired in old CC mice compared to WW controls, as illustrated by lower percent correct choices in three out of the four training blocks (Fig. 1e; genotype: F_1,16_ = 19.19, *p* < 0.001; block: F_3,48_ = 10.98, *p* < 0.001; genotype × block: F_1,16_ = 3.02, *p* = 0.04). The spatial reference memory of CC animals was also impaired, as suggested by the decrease in the time spent exploring the goal arm during the 30-s probe test conducted 24 h after the last training session (Fig. 1e; genotype: F_1,16_ = 7.33, *p* = 0.02). Whilst age-matched WW controls performed significantly above chance level, CC mice did not (33% chance level; WW vs. chance: t_8_ = 3.92, *p* = 0.004; CC vs chance: t_8_ = −0.64, *p* = 0.54). Despite profound deficits in spatial reference learning and memory, basic visual cue discrimination learning was intact in CC mice as shown by the steady and comparable increase in percent correct choices across training blocks in both CC and WW groups (Fig. 1f; genotype: F_1,16_ = 0.22, *p* = 0.64; block: F_9,144_ = 8.06, *p* < 0.001; genotype versus block: F_9,144_ = 0.52, *p* = 0.86).

#### Impaired short-term habituation and spatial working memory in old male rTg4510 CC mice

Short-term habituation and spatial working memory were assessed using three different but complementary maze tasks. rTg4510 mice were first tested in a Y-maze spontaneous continuous alternation task. CC performance was similar to that of WW controls and significantly above alternation chance level (Fig. 1g; genotype: F_1,16_ = 1.31, *p* = 0.27; WW vs. chance: t_8_ = 11.46, *p* < 0.001; CC vs. chance: t_8_ = 7.81, *p* < 0.001). Mice were then tested in a Y-maze spontaneous spatial novelty preference task. CC mice performed poorly compared to WW controls, with a lower and close-to-chance novelty discrimination ratio (Fig. 1h; genotype: F_1,15_ = 33.27, *p* < 0.001; WW vs. chance: t_8_ = 9.93, *p* < 0.001; CC vs. chance: t_7_ = 1.96, *p* = 0.09). Mice were finally tested in a T-maze discrete-trial rewarded alternation task. CC mice were again impaired relative to WW controls, with alternation performance no different than chance level (Fig. 1i; genotype: F_1,12_ = 135.41, *p* < 0.001; WW vs. chance: t_7_ = 35.52, *p* < 0.001; CC vs. chance: t_5_ = 0.00, *p* = 1.00). Pathology at this time point was so extensive that no CC animal was able to perform above the chance level in T-maze performance, and all but one CC mice displayed a robust hyperactive phenotype in open-field locomotor activity.

### Study 2: Validation of selected endpoints in 4- to 12-month-old, doxycycline-treated male rTg4510 mice

#### Missing and excluded data

Five mice (two CC and three CC + dox) were culled due to poor health during the 8-month study; these animals were removed from all behavioural and pathological analyses. One mouse (CC) was excluded from spatial novelty preference analysis as it was able to escape the arena during testing. Five animals (one WW, three CC, and one CC + dox) repeatedly failed to complete more than five trials during T-maze testing and were therefore excluded from T-maze longitudinal analysis. A further two mice (both WW) were removed from T-maze analysis at specific time points where they completed fewer than five trials, but not from the entire analysis.

#### Doxycycline arrested the progression of both tau burden and brain atrophy

**Fig. 2:**
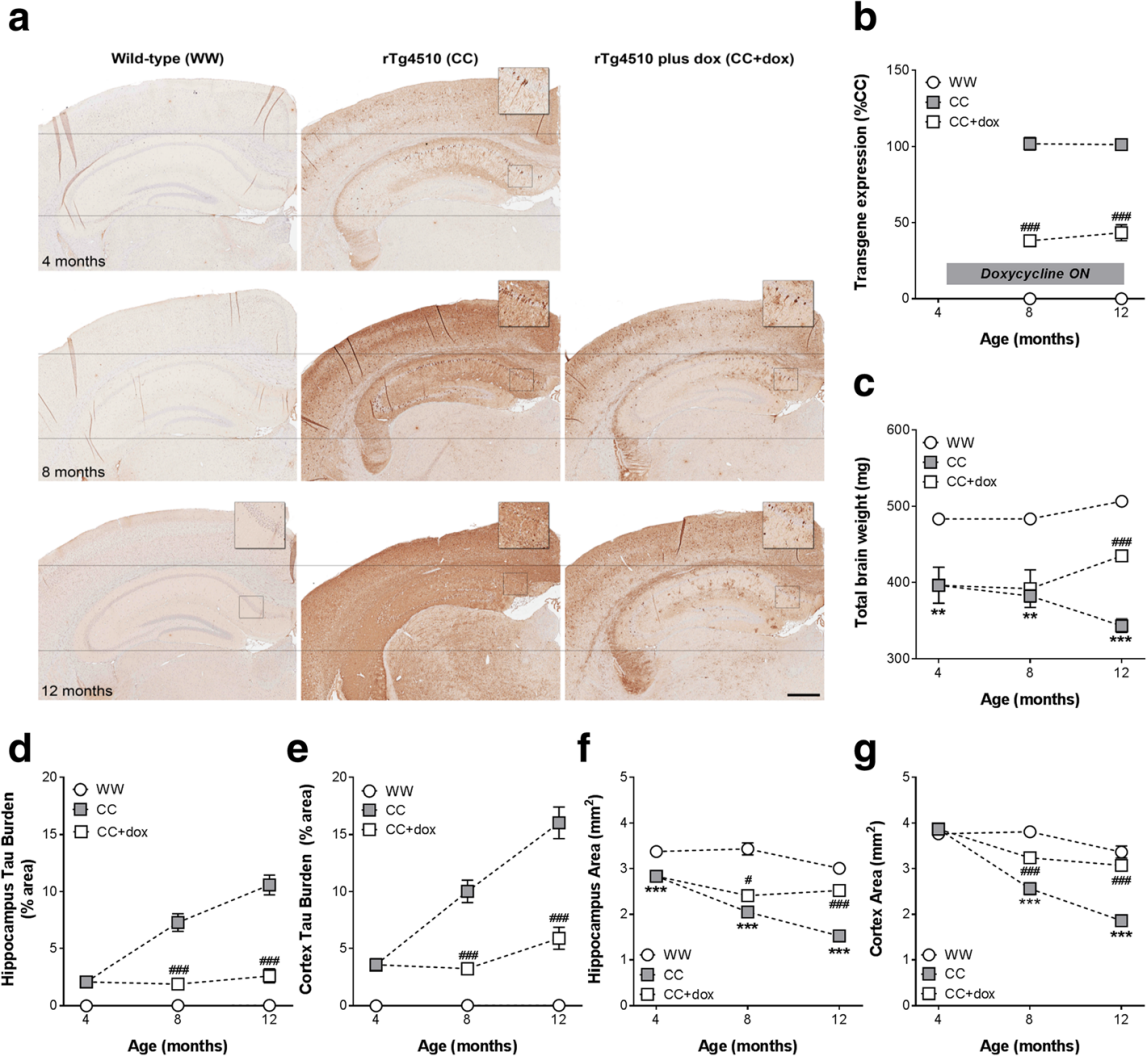
Tau pathology in male rTg4510 mice following doxycycline treatment. Representative immunohistochemical images of 4-, 8-, and 12-month-old male rTg4510 brains (**a**). RT-qPCR revealed a 40 to 50% reduction of tau expression in bi-transgenic rTg4510 (*CC*) mice receiving doxycycline (*dox*) treatment (**b**). Brain weight was decreased in CC mice at all time points, and was attenuated in 12-month-old CC + dox mice (**c**). Increasing levels of tau pathology were observed in both the hippocampus (**d**) and cortex (**e**) of CC mice from 8 months of age. These were normalised following doxycycline treatment. CC mice displayed high levels of atrophy in the hippocampus at all time points (**f**) and in the cortex at 8 and 12 months (**g**); atrophy was prevented by doxycycline treatment. Note that *hashed lines* do not represent longitudinal, repeated assessment of these animals. *Scale bar* = 500 μm. All data are presented as mean ± SEM. *p < 0.05, **p < 0.01, ***p < 0.001, versus wild-type/non-transgenic rTg4510 (*WW*) controls; ^#^ *p* < 0.05, ^##^ *p* < 0.01, ^##^#*p* < 0.001, versus rTg4510 CC mice

Progressive pathological changes were seen in the brains of 4-, 8-, and 12-month-old male rTg4510 mice that appeared to be preserved by doxycycline treatment initiated at 4.1 months (Fig. 2a). This occurred despite only a 40–50% reduction in tau expression compared to CC animals as measured by RT-qPCR analysis (Fig. 2b; 8 months: F_1,40_ = 184.51, *p* < 0.001; 12 months: F_1,42_ = 93.73, *p* < 0.001).

At 4 months of age, tau burden in the hippocampus (2.1 ± 0.2%) and cortex (3.6 ± 0.4%) of CC mice was relatively low (Fig. 2d and e). Signs of brain atrophy were apparent at this stage; whole brain weight was significantly reduced, which coincided with reductions in hippocampal and cortical area (Fig. 2c, f, and g; brain weight: F_1,25_ = 8.92, *p* = 0.006; hippocampus size: F_1,25_ = 42.80, *p* < 0.001; cortex size: F_1,25_ = 0.57, *p* = 0.46). By 8 and 12 months of age, significant increases in tau burden were observed in both the hippocampus (7.3 ± 0.6% and 10.6 ± 0.7%, respectively) and cortex (10.0 ± 0.7% and 16.0 ± 1.1%, respectively). Widespread atrophy was also observed; brain weight was again smaller in 8-month CC mice, and appeared to decrease further at the later time point of 12 months (Fig. 2c; 8 months: F_2,42_ = 7.93, *p* < 0.001; 12 months: F_2,42_ = 101.85, *p* < 0.001). Progressive decreases in size were observed in both hippocampal (Fig. 2f; 8 months: F_2,40_ = 29.33, *p* < 0.001; 12 months: F_2,42_ = 74.44, *p* < 0.001) and cortical (Fig. 2g; 8 months: F_2,42_ = 22.92, *p* < 0.001; 12 months: F_2,42_ = 61.36, *p* < 0.001) areas compared to WW controls.

Despite only moderate levels of transgene suppression at 8 and 12 months, hippocampal (1.9 ± 0.6% and 2.6 ± 0.7%, respectively) and cortical (3.2 ± 0.7% and 5.9 ± 1.1%, respectively) tau burden were significantly decreased in CC + dox mice compared to CC controls (Fig. 2d and e; hippocampus, 8 months: F_2,40_ = 31.96, *p* < 0.001, 12 months: F_2,42_ = 53.16, *p* < 0.001; cortex, 8 months: F_2,42_ = 43.43, *p* < 0.001, 12 months: F_2,42_ = 42.20, *p* < 0.001), levels that were largely comparable to those observed at 4 months. Doxycycline also prevented further brain atrophy, as illustrated by the significant differences in CC + dox vs. CC brain weight (Fig. 2c; 8 months: F_2,42_ = 7.93, *p* = 0.72; 12 months: F_2,42_ = 101.85, *p* < 0.001), hippocampus area (Fig. 2f; 8 months: F_2,40_ = 29.34, *p* = 0.02; 12 months: F_2,42_ = 74.44, *p* < 0.001), and cortex area (Fig. 2g; 8 months: F_2,42_ = 22.92, *p* < 0.001; 12 months: F_2,42_ = 61.36, *p* < 0.001).

#### Doxycycline prevented further decline in behavioural and cognitive function

The pattern of changes observed from 4 to 8 and 12 months was similar across all four behavioural assays. The performance of male rTg4510 CC mice progressively worsened over time, reaching levels similar to those observed in 12- to 15-month-old CC mice during study 1. Doxycycline treatment from 4.1 months prevented further functional decline and showed trends towards reversing initial behavioural deficits.

**Fig. 3:**
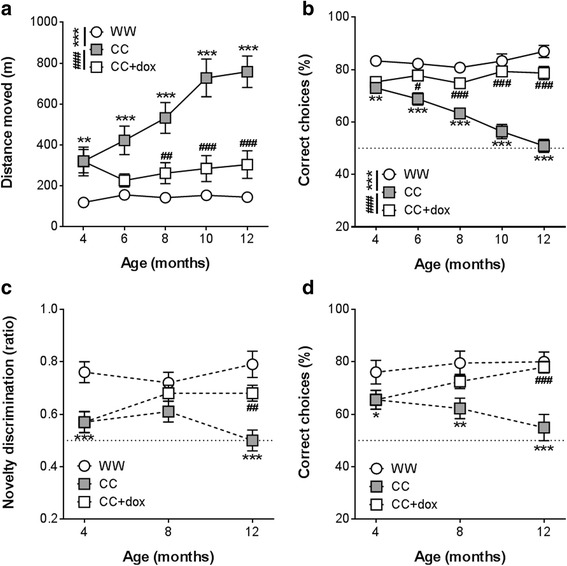
Behavioural alterations in male rTg4510 mice following doxycycline treatment. Locomotor activity revealed bi-transgenic rTg4510 (*CC*) mice to be hyperactive at 4 months and become increasingly more active as they age, whilst CC + doxycycline (*dox*) mice remained stable at levels similar to those observed prior to starting doxycycline treatment (**a**). T-maze rewarded alternation task accuracy was significantly poorer in CC mice at 4 months of age, and declined progressively until reaching chance levels at 12 months. Doxycycline treatment significantly improved performance; CC + dox mice made significantly more correct choices than CC mice from 6 months onwards (**b**). CC mice were impaired in novelty discrimination, as assessed in a spatial novelty preference Y-maze task. CC + dox mice showed improved novelty discrimination compared to CC at 12 months (**c**). Spatial learning was assessed using acquisition of an aversively-motivated Y-maze; CC mice made significantly fewer correct choices over 40 trials than WW mice, and further decreases were seen with age. Doxycycline treatment improved performance, with CC + dox mice making more correct choices than CC at 8 and 12 months (**d**). Note that *hashed lines* do not represent longitudinal, repeated assessment of these animals. All data presented as mean ± SEM, *dotted horizontal li*nes denote chance level. *p < 0.05, **p < 0.01, ***p < 0.001, versus wild-type/non-transgenic rTg4510 (*WW*) controls; ^#^ *p* < 0.05, ^##^ *p* < 0.01, ^###^ *p* < 0.001, versus rTg4510 CC mice

A progressive increase in open-field locomotor activity was observed over time in CC mice, whilst activity in WW littermates remained stable (Fig. 3a; group: F_2,108_ = 31.09, *p* < 0.001; age: F_4,73_ = 19.80, *p* < 0.001; group × age: F_8,99_ = 6.16, *p* < 0.001). CC mice were hyperactive from 4 months of age (*p* = 0.003 versus WW) and became progressively more active at later time points (6–12 months: all *p* < 0.001, versus WW). Doxycycline treatment prevented further increases in locomotor activity in CC mice, with CC + dox mice significantly less active than non-dox-treated counterparts across the 8- to 12-month age period (8–12 months: all *p* < 0.001, versus CC).

T-maze rewarded alternation performance was also progressively impaired in CC mice (Fig. 3b; group: F_2,85_ = 41.47, *p* < 0.001; age: F_4,65_ = 3.99, *p* = 0.006; group × age: F_8,88_ = 6.60, *p* < 0.001). Significant deficits were present at 4 months in CC mice which declined to chance level by 12 months (4–12 months: all *p* < 0.001, versus WW). In contrast, WW mice exhibited stable performance over this time period, while CC + dox mice displayed significantly elevated performance from 6 months of age (6 months: *p* = 0.03; 8–12 months: *p* < 0.001, CC + dox versus CC).

Spatial novelty discrimination in 4-month-old CC mice was impaired compared to WW controls (Fig. 3c; group: F_1,25_ = 14.16, *p* < 0.001); CC performance was no different than chance level. At 8 months, however, no significant difference was observed between all three groups (WW, CC, and CC + dox group: F_2,42_ = 2.17, *p* = 0.13). Finally, at 12 months, novelty discrimination performance of CC mice was again impaired compared to the other two groups of mice (group: F_2,42_ = 11.86, *p* < 0.001). Planned comparisons revealed a significant decrease in novelty discrimination in CC animals (*p* < 0.001, versus WW), which was attenuated by doxycycline treatment (*p* = 0.002, CC + dox versus CC).

Spatial reference learning and memory declined over time in CC mice (Fig. 3d; 4 months: F_1,25_ = 5.05, *p* = 0.03; 8 months: F_2,42_ = 6.21, *p* = 0.004; 12 months: F_2,41_ = 19.32, *p* < 0.001, versus WW), with performance at levels no different than chance by 8 months of age. Treatment with doxycycline from 4.1 months was able to prevent this decline at both 8 and 12 months (8 months: F_2,42_ = 6.21, *p* = 0.017; 12 months: F_2,41_ = 19.32, *p* < 0.001, versus CC), effectively restoring performance to levels that were not significantly different to those of WW animals.

#### Relationships between measures of pathology and/or behaviour

**Fig. 4:**
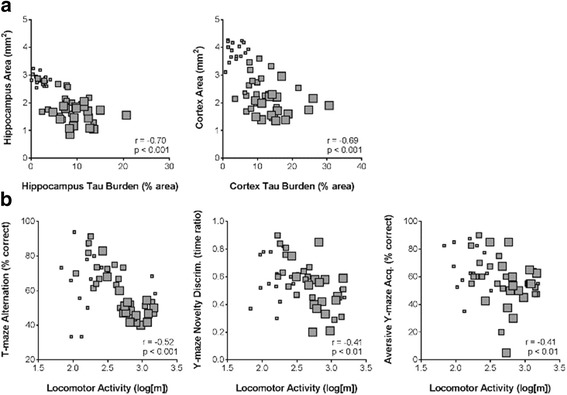
Relationships between measures of pathology or behaviour in male rTg4510 CC mice. Strong negative correlation was observed between measures of tau burden and atrophy in both the hippocampus and cortex (**a**). Open-field locomotor activity was correlated with all three other behavioural assays: T-maze rewarded alternation, Y-maze spatial novelty preference, and the acquisition of the aversive Y-maze spatial reference memory task (**b**). *Small squares* = 4 months; *medium squares* = 8 months; *large squares* = 12 months

**Fig. 5:**
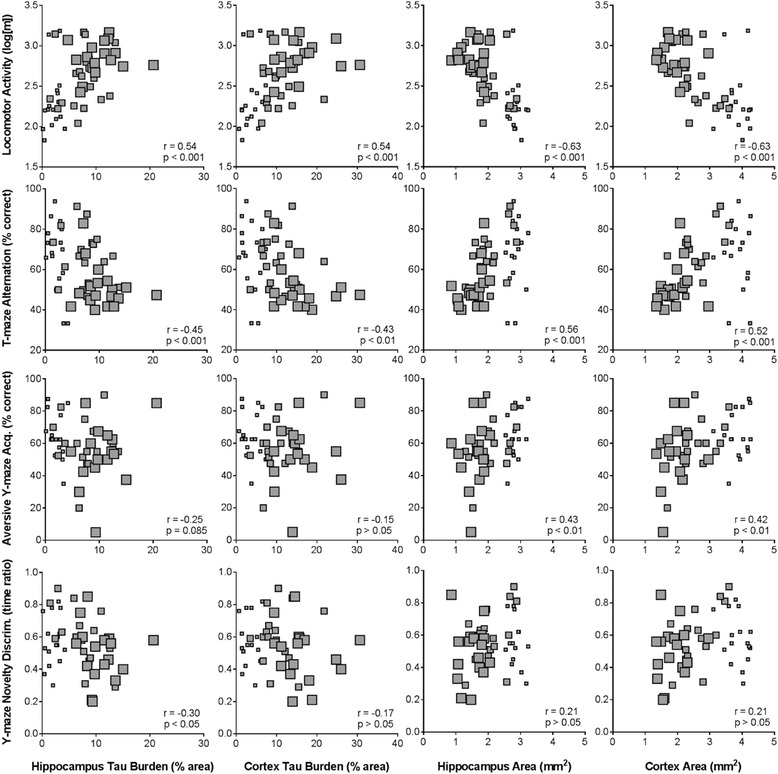
Relationships between measures of pathology and behaviour in male rTg4510 CC mice. Locomotor activity and T-maze performance were strongly related to all pathological endpoints. Aversive Y-maze spatial learning also correlated with atrophy in both the hippocampus and cortex, which was not the case for Y-maze novelty preference. *Small squares* = 4 months; *medium squares* = 8 months; *large squares* = 12 months

Spearman’s rank correlation analyses of the multiple study endpoints revealed strong relationships between measures of tau pathology and brain atrophy in CC mice, but not CC + dox mice (data not shown). In CC mice, hippocampal and cortical tau burden were negatively correlated with hippocampal and cortical area, respectively (Fig. 4a; hippocampus: *r* = −0.70, *p* < 0.001; cortex: *r* = −0.69, *p* < 0.001). Moderate relationships were also observed between various behavioural endpoints. Open-field locomotor activity correlated with all three other behavioural endpoints: T-maze rewarded alternation (Fig. 4b; *r* = −0.52, *p* < 0.001), Y-maze novelty preference (r = −0.41, *p* = 0.01), and aversive Y-maze acquisition (*r* = −0.41, *p* < 0.01). CC mice also showed strong correlations between behavioural and pathological outcomes (Fig. 5). Locomotor activity and T-maze alternation both correlated strongly with all measures of pathology investigated. Aversive Y-maze acquisition correlated with hippocampal and cortical area, but Y-maze novelty preference did not.

## Discussion

The present study aimed to extend on the initial phenotypic characterisation of the rTg4510 mouse model and, in doing so, to define a common experimental approach to selection and validation of the most relevant cognitive and behavioural endpoints. Out of the seven behavioural assays tested at a late pathological stage, four were sensitive to tau pathology. Performance in these assays was shown to worsen progressively with age, to correlate well with developing tau pathology, and to be responsive to experimental modulation of tau expression by doxycycline treatment. The confluence of these three characteristics further illustrated that functional measures such as behavioural performance can be a meaningful proxy for underlying pathology, without the need for invasive measures.

The first study assessed 12-month-old male rTg4510 mice across a range of behavioural tasks to determine which would be sensitive to tau pathology. By this time point, bi-transgenic rTg4510 (CC) mice displayed considerable tau burden and brain atrophy, the extent of which was broadly comparable to that reported previously [18]. CC mice exhibited striking levels of locomotor hyperactivity, profound stereotypy/circling behaviours, and yet intact balance and motor co-ordination. The propensity to become increasingly hyperactive and stereotyped with age is a common characteristic of tau transgenic models [19–21] and needs to be considered when accounting for other behavioural impairments. For instance, male CC mouse performance was no better than chance in both T-maze rewarded alternation and Y-maze novelty preference tasks, though Y-maze continuous alternation performance was just as good as wild-type (WW) mice. Such apparent discrepancy between three tasks of short-term habituation and working memory are likely driven by stereotypy. Circling behaviour may indeed promote or prevent arm alternation respectively in continuous versus discrete-trial alternation paradigms. Several other groups have described behavioural deficits in rTg4510 mice, predominantly in the water maze, but rarely take into consideration how these effects might primarily be driven by stereotypy rather than cognitive dysfunction [21]. Also of interest in this study was that male CC mice could acquire a non-spatial visual cue discrimination maze task as effectively as WW controls. This highlights the ability of the animals to overcome stereotypy in certain scenarios and suggests that some aspects of the rTg4510 CC mouse behavioural repertoire are still intact even at this advanced pathological stage. This stands in contrast to previous findings of impairments in visible platform water maze performance in rTg4510 CC mice [16, 22], and suggests that not all tests of visual capacity may offer concordant outcomes. In conclusion, cognitive and behavioural deficits were observed in four out of the seven assays used. Open-field locomotor activity and T-maze alternation measured CC-related deficits of large effect size and lend themselves well to high-throughput longitudinal studies. Aversive Y-maze spatial reference memory and Y-maze spatial novelty preference tests were also capable of detecting CC deficits. However, these assays are of lower throughput and may evoke confounding practice effects after repeated testing; these assays are therefore more usefully employed in cross-sectional study designs.

In the second study, the four nominated behavioural assays from step 1 were used with the aim of tracking progressive tau-related functional decline from 4 to 12 months of age. Doxycycline was administered from 4.1 months, with the hypothesis that this would prevent further progression of tau pathology, and this in turn would preserve functional capacity. The regional distribution of tau pathology was consistent with that previously observed [15, 23], reflecting the use of the calcium/calmodulin-dependent protein kinase II as a promoter for human tau transgene expression in these animals. Tau burden was apparent at 4 months and increased in an age-dependent manner. The progressive increase in tau pathology was associated with brain atrophy at 8 and 12 months, believed to be a result of synaptic degradation and neuronal loss [15, 18, 24]. Early research suggested that whilst doxycycline suppression of transgene expression was able to prevent further neuronal loss, tau burden continued to accumulate [18]. The present findings were, however, in line with recent literature [24–27], and suggested that despite only moderate levels of tau transgene suppression (i.e. 50%) no further tau accumulation and atrophy were observed once doxycycline treatment was initiated. This level of transgene suppression is lower than previously reported by other groups [14]. This is likely due to the food restriction protocol used in this study, where animals would have received less doxycycline than they would have if they were kept on an ad libitum diet and likely were subject to a lower exposure of the drug as a result. Nonetheless, the fact that only a partial suppression of tau expression by doxycycline was able to modulate subsequent progression of tau pathology and normalise behaviour provides encouraging support that potential clinical therapies do not necessarily have to be maximally effective to exhibit functionally beneficial effects.

Locomotor activity and rewarded T-maze alternation performance at 4 months worsened progressively until 12 months, at which point deficits were of similar magnitude to that observed in the first study, providing an internal replication of the dataset. Progressive decline of function was also observed in the aversive Y-maze spatial reference memory task, and was rescued by doxycycline as previously reported elsewhere in the water-maze task [14, 15, 21, 28]. Doxycycline treatment effects were bi-directional, preventing both the increase in locomotor activity and the decrease in performance in all three other maze tasks. Such bi-directionality of effects lends weight to the interpretation that doxycycline was truly normalising behaviour. This is an important point, as drug studies often report a restorative effect in only one assay and one direction. Only by combining multiple functional endpoints from tests with different ancillary demands can confidence be raised on the meaningfulness of the observations. Other groups are beginning to take this into consideration when testing therapeutic interventions (e.g. [29]). It is also important to highlight that this work only involved male rTg4510 mice. Sex differences have been reported by other groups [16]; female rTg4510 mice have been shown to develop tau pathology at an earlier age and more aggressively than males. An important next step will be to validate the present findings in female mice.

Correlation analyses revealed that pathological and behavioural endpoints were closely related in CC mice. Locomotor activity and T-maze alternation performance displayed moderate to strong relationships with both measures of pathology, i.e. tau burden and atrophy. Such behavioural measures can therefore be taken as indicative of the level of underlying regional pathology and may offer a longitudinal, non-invasive, and high-throughput in-vivo biomarker of progression (and suppression) of tau pathology. Correlations may ultimately be investigated further with newer pre-clinical imaging techniques such as tau positron emission tomography (PET) tracers [30, 31] and multi-parametric magnetic resonance imaging (MRI) [24] that would allow longitudinal assessment of underlying pathology. Such imaging techniques also offer the ability to investigate key features of AD such as atrophy [24], white matter changes [32], and cerebrovascular reactivity [33]. Pairing longitudinal imaging techniques with the behavioural work described here could potentially provide powerful insight into the relationship between functional decline and morphological alterations within diseased brains as the pathology develops.

Given the applied nature of the current work, it is important to comment on how these findings may fit into the bigger picture of AD drug discovery. All pre-clinical models have limitations that should be acknowledged and accounted for wherever possible. Several hundred interventions have been reported to normalise pathological and/or behavioural endpoints in AD mouse models [2, 11–13], with some of these findings reported in rTg4510 mice [26, 34–36]. It is highly unlikely that any one of the many existing mouse models of AD possesses complete predictive validity. Just as AD patients exhibit various degrees of regional pathological burden and diverse functional alterations [37], AD mouse models also differ in these parameters and may display limited correspondence with human AD canonical Braak staging [38]. Mouse models can also present with pathological and functional artefacts as a consequence of their construction. For instance, the tetracycline transactivator (TTA) used to control tau transgene expression in rTg4510 mice may be linked to developmental and functional abnormalities [39–41]. Since no model is perfect, hypothesis testing in multiple models is always preferable as it offers increased confidence of drug effect via convergent validation [42–44].

## Conclusions

The two-step experimental approach used in this study provides a detailed cognitive and behavioural profiling of male rTg4510 mice, with careful matching to pathological progression. Only four of the seven behavioural assays initially tested produced robust deficits in male rTg4510 mice, highlighting the importance of careful selection and validation of functional endpoints prior to testing. This approach can easily be applied to increase our understanding of other transgenic mouse models of AD. Ultimately, better understanding of the nature of functional and pathological decline in such models of interest may lead to better study design, and in turn improve confidence in pre-clinical validation of novel, emerging therapeutic interventions. This will hopefully lead to more robust, translatable packages enhancing chances of clinical success in the future.

## Abbreviations

AD: Alzheimer’s Disease
ANOVA: Analysis of variance
CC: Bi-transgenic rTg4510 mice
Dox: Doxycycline
IHC: Immunohistochemistry
ITI: Inter-trial interval
RT-qPCR: Reverse transcription quantitative polymerase chain reaction
ROI: Region of interest
rTg4510: rTg(tet-o-TauP301L)4510
WW: Wild-type/non-transgenic rTg4510 mice
